# Precision Nursing Guided by IKAP Theory for Volume Management in Elderly Patients with Chronic Heart Failure: A Retrospective Study

**DOI:** 10.12669/pjms.42.3.14435

**Published:** 2026-03

**Authors:** Lulu Ma, Liang Tao, Yulan Liu, Yi Xu, Si Huang

**Affiliations:** 1 Lulu Ma Department of Cardiovascular 2, Xiangtan City Central Hospital, Xiangtan, Hunan Province 411100, P.R. China; 2 Liang Tao Department of Cardiovascular 2, Xiangtan City Central Hospital, Xiangtan, Hunan Province 411100, P.R. China; 3 Yulan Liu Department of Cardiovascular 2, Xiangtan City Central Hospital, Xiangtan, Hunan Province 411100, P.R. China; 4 Yi Xu Department of Cardiovascular 2, Xiangtan City Central Hospital, Xiangtan, Hunan Province 411100, P.R. China; 5 Si Huang Department of Cardiovascular Intensive Care, Xiangtan City Central Hospital, Xiangtan, Hunan Province 411100, P.R. China

**Keywords:** Elderly Chronic Heart Failure, IKAP Theory, Precision Nursing, Volume Management

## Abstract

**Objective:**

To explore the efficacy of precision nursing based on the Information-Knowledge-Attitude-Practice (IKAP) theory in volume management of elderly patients with chronic heart failure (CHF).

**Methodology:**

This single-center retrospective study included 115 elderly patients with CHF admitted between May 2022 and March 2025. Patients received either routine nursing (control group, n=56) or IKAP-guided precision nursing for volume management (study group, n=59). Key outcomes included self-care/volume-management ability, cardiac function, volume management related indicators, and complications.

**Results:**

After intervention, self-care/volume-management scores improved in both groups and were higher in the study group (all P<0.05). The study group showed better echocardiographic parameters (higher LVEF and lower LVEDD/LVESD) and more favorable volume-management–related indicators than the control group (all P<0.05), with fewer complications (3.38% vs 14.29%, P<0.05).

**Conclusion:**

IKAP-guided precision nursing may improve self-care for volume management and cardiac function and reduce complications in elderly patients with CHF, supporting a structured, theory-driven nursing approach in routine practice.

## INTRODUCTION

With the gradually aging global population, the number of elderly patients with chronic heart failure (CHF), a severe and terminal stage of cardiovascular disease, is on the rise.^1^,^2^ The prevalence of CHF in people over 60 years old is as high as 6%-10%, and the disease is more complex and associated with a worse prognosis due to the decline of multiple organ functions and multiple comorbidities in the elderly population of patients.^3^,^4^ Volume management is a crucial component of CHF treatment, and includes maintaining patients’ fluid balance, reducing cardiac load, and lowering readmission rates. However, the effectiveness of volume management is affected by poor compliance and insufficient self-management ability in elderly patients.^5^,^6^

At present, clinical volume management for elderly CHF patients is carried out through routine nursing models that focus on disease knowledge education and treatment cooperation guidance, but lack in-depth exploration of the internal mechanisms underlying patients’ behavioral changes and are not personalized.^7^,^8^ Elderly CHF patients often fail to effectively implement measures such as salt or water restriction and weight monitoring due to insufficient knowledge and cognition, lack of self-management skills, and weak health beliefs, which, in turn, affect the effectiveness of volume management.^9^,^10^

The main principle of the Information-Knowledge-Attitude-Practice (IKAP) theory is that the formation of an individual’s health behaviors is a continuous process involving information acquisition, knowledge accumulation, attitude transformation, and behavior change.^11^ Applying the IKAP theory to nursing practice enables the development of personalized intervention measures by accurately assessing patients’ needs at each stage, thereby enabling effective guidance from knowledge transfer to behavior change.^12^ However, the effectiveness of this theory for volume management in elderly patients with chronic heart failure (CHF) remains unclear. This study aimed to assess the efficacy of a precise IKAP theory-based nursing plan in the volume management of elderly CHF patients. The results may provide new directions and a scientific basis for improving the nursing quality and prognosis of elderly CHF patients.

## METHODOLOGY

The clinical data of 115 elderly patients with CHF, admitted to Xiangtan City Central Hospital from May 2022 to March 2025, were retrospectively selected. Patients were identified from the hospital electronic medical record system within the predefined study window and were screened in chronological order according to the prespecified inclusion and exclusion criteria. Among them, 56 patients received routine nursing and were assigned to the control group, and 59 patients received precision nursing based on the IKAP theory and were named as the study group. Group classification was based on the nursing management model actually implemented in routine care during hospitalization, rather than any subjective allocation by the investigators. In this study, “precision nursing” refers to an IKAP-guided, individualized volume-management nursing strategy that integrates tailored information delivery, knowledge reinforcement, attitude/motivation support, and behavior implementation with structured follow-up.

### Ethical approval:

The Medical Ethics Committee of Xiangtan Central Hospital approved this study with the number 2025-09-011, Date: September 22, 2025. The requirement for informed consent was waived by the ethics committee due to the retrospective design and minimal risk to participants. All data were extracted from existing medical records and were de-identified prior to analysis to protect patient privacy.

### Inclusion Criteria:

- Meet the diagnostic criteria for CHF.^13^
- Aged ≥ 60 years.
- Stable condition (New York Heart Association (NYHA) cardiac function classification of Grade II-IV).
- Possess basic communication and comprehension abilities, able to express feelings independently, and cooperative in completing questionnaires.
- No severe cognitive impairment.
- Complete clinical data.

### Exclusion Criteria:

- Major comorbidities that may affect the outcome of volume management, such as severe liver and kidney failure, malignant tumors, and hematological diseases.
- Mental illnesses (such as schizophrenia, mania) or consciousness disorders.
- Occurrence of acute heart failure or a history of special treatments (such as heart transplantation, mechanical circulatory support) within one month before enrollment.
- History of alcohol or drug dependence, or severe visual/hearing impairment that affects information reception and behavior learning.
- Poor compliance, unable to record daily weight, fluid intake and output, and dietary status as required.

### Intervention plans:

### Routine Nursing:

The regimen combined the following:

### Basic Nursing:

Patients were assisted with daily living activities, a clean and quiet ward environment with appropriate temperature (22-24°C) and humidity (50%-60%) was maintained. Patients were helped to turn over; care was taken to prevent pressure ulcers and pulmonary infections by frequent repositioning and percussion.

### Condition Observation:

Patients’ vital signs, including heart rate, blood pressure, respiratory rate, and blood oxygen saturation, were closely monitored. Body weight was measured and recorded daily; symptoms such as dyspnea, cough, expectoration, and edema were monitored. All changes in symptoms were tracked, and abnormalities were promptly reported to doctors.

### Medication Nursing:

Necessary medications were administered accurately in strict accordance with the doctor’s orders. The name, dosage, administration method, effect, and adverse reactions of medications were explained to patients. The use of drugs such as diuretics and cardiotonics was specifically emphasized. The therapeutic effect and adverse reactions (such as electrolyte disorders like hypokalemia and hyponatremia) after medication were monitored.

### Dietary Nursing:

Dietary nursing consisted of counseling patients to follow a low-salt diet with a daily salt intake of ≤3 g. Fluid intake was restricted, and daily water consumption was adjusted according to patients’ cardiac function (generally controlled at 1500-2000ml). The implementation of restrictions was supervised, and patients were encouraged to eat easily digestible foods rich in vitamins and protein to maintain balanced nutrition.

### Activity Guidance:

Patients were advised on activities based on their cardiac function classification. Patients with NYHA Class III were guided to alternate between rest and activity, with activity intensity that does not cause fatigue, palpitations, or dyspnea. Patients with NYHA Class II were instructed to mainly stay in bed and were allowed to perform in-bed activities. Patients with NYHA Class IV maintained absolute bed rest.

### Precision Nursing Based on the IKAP Theory:

In addition to the basic nursing regimen, patients received:

### (Information, Information Support):

### Disease Knowledge Education:

Illustrated manuals on chronic heart failure (CHF) and volume management were developed. The manuals covered content such as disease etiology, symptoms, treatment methods, the importance of volume management (such as the impact of excessive fluid intake on cardiac load), and the significance of weight monitoring. The manuals were distributed to patients and their families. Centralized health lectures were conducted once a week. Primary nurses or specialist doctors provided information on the disease in plain language, discussed the consequences of improper volume management, leading to disease deterioration, using real cases, and set up a Q&A session to address questions from patients and their families.

### Personalized Information Push:

Based on patients’ educational background and cognitive level, personalized health knowledge was “pushed” via WeChat official accounts, short videos, etc. For patients with a lower educational background, this included animated videos, explaining key points of volume management. For patients with a higher educational background, professional literature interpretations, the latest research progress, and other content were provided. Patients’ health records were established to document basic information, disease status, medication use, etc., and targeted health reminders were set based on the record information, such as medication time, follow-up appointment time, and dietary precautions.

### K (Knowledge, Knowledge Acquisition):

### Knowledge Assessment and Enhancement:

Within 24 hours of patients’ admission, their understanding of CHF and volume management knowledge was established using a self-designed questionnaire. Personalized knowledge enhancement plans were developed based on the assessment results. One-on-one knowledge explanations were provided to patients with poor knowledge mastery, using auxiliary tools such as pictures and models to help them understand. Patients with good knowledge mastery were encouraged to share their own experiences and further expand related knowledge (such as CHF prevention measures).

### Interactive learning:

Group discussion activities were organized for patients and their families, focusing on common issues in volume management, such as “how to accurately record daily water intake” and “how to adjust diet when edema occurs” to share experiences and insights. Scenario simulation teaching was conducted. Different volume management scenarios (such as sudden weight gain, severe thirst in patients) were set up. Patients and their families were asked to simulate response measures, while nurses provided on-site guidance and corrections.

3) A (Attitude, Attitude Transformation)

### Psychological support and Motivation stimulation:

A good nurse-patient relationship was established. Primary nurses communicated with patients regularly to understand their psychological status, and provided personalized psychological counseling for patients’ concerns (such as worrying about disease prognosis, fearing dietary and activity restrictions). Patients with well-controlled conditions and active cooperation in treatment and nursing were invited to share their experiences to stimulate patients’ enthusiasm and confidence in actively participating in volume management and to help them establish a correct attitude towards treatment.

### Family support promotion:

Health education was provided for patients’ family members, emphasizing the importance of family support in volume management, and guiding family members on how to assist patients in dietary control and weight monitoring. Family members were encouraged to participate in the nursing process, such as recording daily diet and water intake with patients and supervising patients’ activities. Frequent seminars for family members were organized to exchange nursing experiences and address any problems.

### 4) P (Practice, Behavior Implementation):

### Precise volume monitoring:

The nursing team trained patients to measure body weight using a standardized protocol (such as early morning fasting, after urination, wearing the same clothing) and to document the measurements accurately. In cases of significant weight increase within a short period (such as more than 1kg in one day), patients were instructed to contact medical staff immediately. Patients were taught to record 24 hours fluid intake and output, including water intake from drinking, water content in food, urine volume, and sweat volume, using a unified record form. Nurses regularly reviewed records and provided guidance and corrections.

### Precise dietary management:

Personalized dietary plans were developed for patients based on their weight, cardiac function status, and activity level. Food models and pictures were used to intuitively show patients the salt and water content of different foods, helping them choose appropriate foods. For patients with comorbidities such as diabetes, dietitians were invited to develop comprehensive dietary plans to ensure balanced nutrition while meeting volume management needs. Dietary implementation was regularly evaluated and adjusted.

### Activity and Rest guidance:

Individualized activity plans (such as walking, Tai Chi) for patients were developed based on their NYHA classification and physical condition, with detailed arrangements for exercise intensity and duration. Patients were advised to schedule rest periods appropriately, maintain adequate sleep, and avoid overexertion and emotional stress. Activity volume and sleep quality were monitored through smart bracelets and other devices, and activity and rest plans were adjusted based on the data.

### Follow-up and Transitional care:

After discharge, regular follow-ups were conducted via phone calls and WeChat, with the frequency of once each in the 1st week, 2nd week, 1st month, and 3rd month after discharge.

### Standardization and delivery:

All patients in the intervention group received a standardized core package for volume management guided by the IKAP framework. The core components included baseline assessment within 24 hours of admission, structured education at least once per week during hospitalization, daily bedside reinforcement with review of weight and intake-output records, and use of standardized self-monitoring tools with predefined alert thresholds. Each structured education session typically lasted approximately 15 to 20 minutes, and bedside reinforcement was incorporated into routine nursing rounds. Post-discharge follow-up was scheduled at one week, two weeks, one month, and three months. The intervention was delivered by a dedicated heart failure nursing team under a unified protocol, and documentation was standardized using the same monitoring forms. Supervisory checks were conducted regularly to support implementation fidelity. Individual tailoring was limited to the delivery format and emphasis of education according to patient literacy and needs, while the core components and schedule remained consistent across participants.

### Observation indicators:

The following indicators were collected for all patients:

Self-volume management ability, assessed using the Self-Care of Heart Failure Index (SCHFI) before and after intervention. The index includes three dimensions: self-management confidence, self-management maintenance, and self-management ability, with scores ranging from 0 to 100. A higher score indicates stronger self-volume management ability.

Cardiac Function, evaluated via ultrasound examination before and after intervention to measure left ventricular end-diastolic diameter (LVEDD), left ventricular end-systolic diameter (LVESD), and left ventricular ejection fraction (LVEF).

Self-volume management effect was evaluated using volume-management–related indicators extracted from routine clinical records, including extravascular lung water index (ELWI), cardiac output, and vascular volume. ELWI reflects pulmonary fluid burden and serves as a quantitative surrogate of pulmonary congestion, whereas vascular volume reflects intravascular filling status and overall circulatory volume.

Complication rate for complications such as atrial fibrillation, embolism, cardiogenic shock, and arrhythmia.

### Statistical analysis:

Data were analyzed using SPSS 26.0. Measurement data were expressed as mean ± standard deviation (\bar{x}\pm s) and compared using the t-test. Count data were expressed as frequency and composition ratio (%) and compared using the χ² test. A P value < 0.05 was considered statistically significant.

## RESULTS

There were no statistically significant differences in general information (such as gender, age, disease duration, NYHA classification, body mass index, education level, per capita monthly household income, and living status) between the two groups (P>0.05), indicating comparability (Table-I).

**Table-I:** Comparison of General Data Between the Two Groups.

Item	Study Group(n=59)	Control Group(n=56)	t/χ^2^	P
***Gender[n(%)]***				
- Male	38(64.41)	39(69.64)	0.356	0.551
- Female	21(35.59)	17(30.36)		
Age(*x̅*±*s*, years)	69.77±6.05	70.28±5.71	0.464	0.643
Course of Disease(*x̅*±*s*, years)	4.12±1.32	4.05±1.56	0.260	0.795
***NYHA stage[n(%)]***				
- Grade II	16(27.12)	18(32.14)	0.532	0.767
- Grade III	24(40.68)	23(41.07)		
- Grade IV	19(32.20)	15(26.79)		
Body Mass Index(*x̅*±*s*, kg/m^2^)	23.05±2.19	22.94±2.35	0.260	0.796
***Education Level[n(%)]***				
- Primary School and Below	35(59.32)	32(57.14)	0.808	0.668
- Middle School	11(18.64)	14(25.00)		
- College and Above	13(22.03)	10(17.86)		
***Per Capita Monthly Household Income[n(%)]***				
≤3000 Yuan	38(64.41)	34(60.71)	0.167	0.682
≤3000 Yuan	21(35.59)	22(39.29)		
***Living Status[n(%)]***				
- Living Alone	8(13.56)	11(19.64)	0.771	0.380
- Not Living Alone	51(86.44)	45(80.36)		

Before the nursing intervention, there were no significant differences in self-management confidence, self-management maintenance, and self-management ability scores between the two groups (P > 0.05). Table-II After intervention, scores on the three dimensions increased in both groups compared with pre-intervention scores, and the study group showed higher scores than the control group (P < 0.05).

**Table-II:** Comparison of Self-volume Management Ability Between the Two Groups (*x̅*±*s*, Scores).

Time	Group	Number of Cases	Self-management Confidence	Self-management Maintenance	Self-management Ability
Before Intervention	Study Group	59	62.72±6.36	65.77±5.28	61.23±7.05
	Control Group	56	64.05±5.40	66.64±6.06	60.99±8.15
	*t*		1.206	0.822	0.169
	*P*		0.230	0.413	0.866
After Intervention	Study Group	59	86.95±7.55	84.80±6.14	87.80±5.17
	Control Group	56	83.08±6.68	81.42±5.95	84.62±4.94
	*t*		2.905	2.995	3.369
	*P*		0.004	0.003	0.001

Pre-intervention values of left ventricular end-diastolic diameter (LVEDD), left ventricular end-systolic diameter (LVESD), and left ventricular ejection fraction (LVEF) were comparable in the two groups (P > 0.05). After intervention, LVEDD and LVESD decreased in both groups, while LVEF increased from pre-intervention levels. Moreover, the indices in the study group were superior to those in the control group (P < 0.05; Table-III).

**Table-III:** Comparison of Self-volume Management Ability Between the Two Groups (*x̅*±*s*).

Time	Group	Number of Cases	Self-management Confidence	Self-management Maintenance	Self-management Ability
Before Intervention	Study Group	59	62.72±6.36	65.77±5.28	61.23±7.05
	Control Group	56	64.05±5.40	66.64±6.06	60.99±8.15
	*t*		1.206	0.822	0.169
	*P*		0.230	0.413	0.866
After Intervention	Study Group	59	86.95±7.55	84.80±6.14	87.80±5.17
	Control Group	56	83.08±6.68	81.42±5.95	84.62±4.94
	*t*		2.905	2.995	3.369
	*P*		0.004	0.003	0.001

Before intervention, there were no significant differences in the extravascular lung water index, cardiac output, and vascular volume between the two groups (P > 0.05). Table-IV post-intervention values of the three indicators in both groups significantly increased and were considerably higher in the study group than in the control group (P < 0.05). The incidence of complications in the study group (3.38%) was significantly (P < 0.05) lower than that in the control group (14.29%), as summarized in Table-V.

**Table-IV:** Comparison of Self-volume Management Effect Between the Two Groups(*x̅*±*s*).

Time	Group	Number of Cases	Extravascular Lung Water Index(ml/kg)	Cardiac Output(L/min)	Vascular Volume(L/min)
Before Intervention	Study Group	59	4.30±0.41	3.09±0.32	0.57±0.09
	Control Group	56	4.19±0.50	3.13±0.29	0.59±0.08
	*t*		1.293	0.701	1.257
	*P*		0.199	0.485	0.211
After Intervention	Study Group	59	8.09±0.73	4.83±0.44	1.25±0.15
	Control Group	56	7.69±0.49	4.09±0.53	1.09±0.13
	*t*		2.574	8.163	6.099
	*P*		0.011	0.000	0.000

**Table-V:** Comparison of complications between the two groups [n (%)].

Group	Number of cases	Atrial fibrillation	Embolism	Cardiogenic shock	Arrhythmia	Total incidence rate
Study group	59	1(1.69)	0(0.00)	0(0.00)	1(1.69)	2(3.38)
Control group	56	2(3.57)	1(1.79)	2(3.57)	3(5.36)	8(14.29)
*χ*²						4.296
*P*						0.038

## DISCUSSION

The results of the study show that IKAP theory-based nursing is associated with improved self-volume management ability, cardiac function, better self-volume management effect, and reduced risk of complications in elderly patients with CHF.

Volume management throughout the entire course of CHF treatment directly affects patients’ prognosis and quality of life.^14^ In agreement with this observation, the results of this study showed that the scores of self-management confidence, self-management maintenance, and self-management ability in the study group were significantly higher than those in the control group (P < 0.05). These results further confirm that precision nursing guided by the IKAP theory can effectively improve patients’ self-volume management ability. The results of this study are consistent with previous research. A study by Gong Y et al.^15^ found that the volume management program based on the IKAP theory, which implements all-round intervention based on information, knowledge, belief, and behavior, can effectively improve the quality of life, weight management ability, and dry body weight compliance rate of heart failure patients, reduce levels of B-type natriuretic peptide level and lower readmission rate during the vulnerable period.

A study by Rogers C et al.^16^ reported that volume management in the elderly CHF patients is challenging due to age-related cognitive decline and insufficient mastery of disease management knowledge. The precision nursing, based on the IKAP theory, conducts progressive interventions through the “information-knowledge-belief-behavior” process. It conveys the core knowledge of volume management (such as controlling fluid intake and the importance of weight monitoring) through diverse formats, including graphic manuals and video explanations. Patients’ understanding of disease management is further strengthened through repeated education and one-on-one Q&A sessions. This nursing approach enhances patients’ self-management competency through methods such as sharing successful cases and family collaborative supervision. It encourages patients to actively and systematically adopt behaviors such as diet and monitoring of intake and output.^17^ In contrast, conventional nursing mainly focuses on the passive implementation of medical orders and simple oral education, lacking a systematic approach and individualized care. Therefore, it is less effective at stimulating patients’ subjective initiative, resulting in limited improvement in self-management.

This study found that while nursing intervention reduced LVEDD and LVESD and increased LVEF in both groups, the improvement in patients in the study group was significantly greater than that in the control group (P < 0.05). The precision nursing based on the IKAP theory, therefore, has a positive effect on optimizing patients’ cardiac function. It is plausible that this nursing mode improves patients’ compliance with volume management, reduces fluid retention, effectively lowers preload, and reduces the burden on the heart. Furthermore, through continuous health guidance, this nursing approach helps patients reasonably adjust diuretic use, optimize their diet, and maintain hemodynamic stability, thereby promoting improvements in cardiac structure and function. In contrast, conventional nursing lacks in-depth guidance and cannot fully meet the individual needs of patients, therefore reducing its effect on cardiac function improvement.^18^ A study by Wang W et al.^19^ found that the continuous nursing based on the IKAP mode for elderly patients with coronary heart disease complicated with heart failure improves patients’ compliance with medical advice, facilitates the recovery of patients’ cardiac function, and thus achieves the goal of improving their quality of life, which is consistent with the view of this study.

A plausible explanation for the greater improvement in LVEF and the greater reduction in LVEDD and LVESD in the intervention group is that IKAP-guided precision nursing strengthens key self-care behaviors and care processes that support decongestion and hemodynamic stability 20-22. Information delivery and knowledge reinforcement may improve patients’ understanding of sodium and fluid restriction and the clinical meaning of daily weight and intake-output monitoring, thereby improving the quality and consistency of self-monitoring.^20^ Attitude and motivation support, together with caregiver involvement, may enhance self-efficacy and treatment adherence, including adherence to prescribed diuretics and other guideline-directed therapies.^21^-^25^ In the practice stage, the use of standardized monitoring records and alert thresholds may facilitate earlier recognition of symptom changes and timely contact with healthcare providers, which may reduce recurrent congestion and repeated decompensation episodes.^20^,^21^,^23^,^26^ Collectively, these behavior and process improvements may plausibly mitigate volume overload and adverse ventricular remodeling, which is consistent with the observed echocardiographic changes.^20^-^22^ Future prospective studies should directly assess adherence, care-process fidelity, and downstream outcomes such as readmission to further validate these pathways.

In this study, the precision nursing based on the IKAP theory was associated with a considerable improvement in extravascular lung water index (ELWI), cardiac output, and vascular volume compared to the standard nursing (P < 0.05). Although ELWI and vascular volume are less commonly reported in nursing research, both parameters provide clinically meaningful information for chronic heart failure volume management. Congestion and volume overload are major drivers of symptom deterioration, decompensation events, and subsequent healthcare utilization.^3^,^13^,^27^ ELWI quantifies pulmonary fluid burden and can help reflect the pulmonary component of congestion, which is closely linked to dyspnea and acute decompensation risk.^28^-^30^ Vascular volume, as a volume-related hemodynamic parameter, reflects intravascular filling status and circulatory volume and can complement symptom-based assessment when characterizing volume status.^31^,^32^ Interpreting these indicators together with improved self-care performance may help explain how IKAP-guided precision nursing supports earlier detection of volume changes and more timely behavioral or treatment adjustment.^20^,^23^,^26^ Nevertheless, measurement and documentation practices may vary across institutions; therefore, future prospective studies should standardize these assessments and further validate their prognostic utility in nursing-led interventions.^27^,^30^

Furthermore, the incidence of complications in the study group (3.38%) was significantly lower than in the control group (14.29%), further reflecting the clinical advantages of precision nursing based on the IKAP theory. Elderly CHF patients are prone to complications such as acute pulmonary edema and electrolyte imbalance due to improper volume management. The precision nursing, based on the IKAP theory, dynamically evaluates patients’ volume status, promptly identifies and corrects misunderstandings in volume management, guides patients to adjust fluid intake based on weight changes, and standardizes their medication. Additionally, with family participation and remote monitoring, this nursing approach ensures comprehensive patient management and effectively reduces the risk of volume-related complications. The results of this study are consistent with previous research^15^ and further demonstrate that behavioral intervention can improve the outcomes of CHF patients more effectively than passive management in conventional nursing.

The IKAP-guided precision nursing program is primarily education- and behavior-oriented and can be delivered as a modular package integrated into routine nursing workflows, with limited additional equipment requirements. From an operational perspective, the main resource inputs are staff time for structured education, reinforcement of self-monitoring, and post-discharge follow-up; however, the observed reduction in complications and improvement in self-management may plausibly translate into lower downstream healthcare utilization, although direct cost data were not collected in this retrospective study and formal cost-effectiveness evaluation is required. Implementation in under-resourced settings may face challenges such as constrained nurse-to-patient ratios, variability in staff training, limited access to digital tools, difficulties maintaining follow-up, and reduced adherence among patients with low health literacy. Practical adaptations may include simplified low-literacy materials, structured caregiver engagement, tiered follow-up that prioritizes high-risk patients, brief group education sessions during hospitalization, and telephone-based follow-up when internet access is limited. Future multi-center prospective studies should incorporate implementation metrics and health-economic analyses to evaluate scalability and real-world value.

This study provides theory-driven real-world evidence by operationalizing an IKAP-guided precision nursing pathway specifically targeting volume management in elderly patients with chronic heart failure. The intervention was structured and potentially reproducible within routine nursing workflows, incorporating standardized self-monitoring, caregiver involvement, and scheduled follow-up. In addition, the outcome assessment was clinically meaningful and multidimensional, including self-care ability, volume-management–related indicators, cardiac function parameters, and complications, which enhances interpretability for nursing practice and supports translation into clinical care.

Future studies should validate these findings using multi-center prospective designs, ideally randomized or quasi-experimental approaches with adequate adjustment for confounders. Longer follow-up is needed to evaluate hard clinical endpoints such as readmission, mortality, and health-related quality of life. Process evaluation is also warranted to examine intervention fidelity, adherence trajectories, and the mechanisms linking IKAP components to behavioral change and clinical outcomes. Finally, health-economic and implementation-science studies should be incorporated to assess scalability, resource requirements, and adaptation strategies, particularly in under-resourced settings.

### Limitations:

Despite these strengths, several limitations should be acknowledged. This single-center retrospective study had a relatively small sample size, which may introduce selection bias and limit the generalizability of the findings. Although baseline characteristics were compared using P values and test statistics, non-significant differences do not necessarily indicate true equivalence in a small retrospective cohort; therefore, residual confounding cannot be fully excluded. Future studies should adopt more robust strategies to assess and control baseline imbalances, including standardized mean differences, propensity score methods, and multivariable adjustment. In addition, the intervention period was relatively short and long-term follow-up outcomes were not available, such as readmission and health-related quality of life. Therefore, multicenter, large-sample prospective studies, preferably with randomized or quasi-experimental designs and extended follow-up, are warranted to confirm the long-term effectiveness of IKAP-guided precision nursing in elderly patients with chronic heart failure.

## CONCLUSION

IKAP-guided precision nursing was associated with improved self-volume management ability, better cardiac function, and more favorable volume-management–related outcomes in elderly patients with chronic heart failure, while reducing the risk of complications. These findings support the clinical value of a structured, theory-driven nursing strategy for volume management in this population. This approach may be particularly suitable for older patients with preserved cognitive and communication ability, adequate motivation for self-monitoring, and strong caregiver or family support, as these factors facilitate sustained adherence and follow-up. Future prospective studies with stratified designs, longer follow-up, and hard clinical endpoints are warranted to confirm subgroup-specific benefits and long-term effectiveness.

### Recommendations:

Future studies should explore integrating the IKAP theory with intelligent wearable devices and mobile medical platforms to enable real-time monitoring and remote intervention in volume management.

### Authors’ contributions:

**LM and LT:** Literature search, study design and manuscript writing.

**YL, YX and SH:** data collection, data analysis and interpretation. Critical Review.

**LM and LT:** manuscript revision and validation and is responsible for the integrity of the study.

All authors have read and approved the final manuscript.
